# Ventriculoperitoneal Shunts Equipped with On-Off Valves for Intraventricular Therapies in Patients with Communicating Hydrocephalus due to Leptomeningeal Metastases

**DOI:** 10.3390/jcm7080216

**Published:** 2018-08-14

**Authors:** Michael C. Burger, Marlies Wagner, Kea Franz, Patrick N. Harter, Oliver Bähr, Joachim P. Steinbach, Christian Senft

**Affiliations:** 1 Dr. Senckenberg Institute of Neurooncology, Goethe University Hospital, 60528 Frankfurt, Germany; oliver.baehr@kgu.de (O.B.); joachim.steinbach@kgu.de (J.P.S.); 2 University Cancer Center Frankfurt (UCT), 60590 Frankfurt, Germany; 3 Institute of Neuroradiology, Goethe University Hospital, 60528 Frankfurt, Germany; marlies.wagner@kgu.de; 4 Department of Neurosurgery, Goethe University Hospital, 60528 Frankfurt, Germany; k.franz@em.uni-frankfurt.de (K.F.); c.senft@med.uni-frankfurt.de (C.S.); 5 Institute of Neurology (Edinger Institute), Goethe University Hospital, 60528 Frankfurt, Germany; patrick.harter@kgu.de; 6 German Cancer Consortium (DKTK), Partner Site Frankfurt/Mainz, 60590 Frankfurt, Germany

**Keywords:** leptomeningeal metastases, hydrocephalus, intraventricular chemotherapy, ventriculoperitoneal shunt

## Abstract

Ventriculoperitoneal shunts equipped with a reservoir and a valve to manually switch off the shunt function can be used for intraventricular injections of therapeutics in patients suffering from a communicating hydrocephalus caused by leptomeningeal metastases. These shunt devices avoid the risk of injecting therapeutics through the distal leg of the shunt system into the intraperitoneal space, which may cause toxicity. Furthermore, regular intraventricular injections of chemotherapeutics help to maintain sufficient concentrations in the ventricular space. Therefore, ventriculoperitoneal shunts equipped with an on-off valve are a useful tool to reliably inject chemotherapeutics into the ventricles. In order to systematically assess feasibility, safety, and efficacy of this procedure, we performed a retrospective analysis of all patients with leptomeningeal metastases who had received a shunt system at our institution. In total, six adult patients had a ventriculoperitoneal shunt equipped with an on-off valve implanted. Out of these six patients, two patients subsequently received intraventricular injections of chemotherapeutics. The configuration of the valve setting and the intraventricular injections were easily feasible in the setting of a neuro-oncology department. The complication of a shunt leakage occurred in one patient following the first intraventricular injection. No extra-central nervous system (CNS) toxicities were observed. In summary, ventriculoperitoneal shunts with on-off valves are useful tools for reliable intraventricular administration of therapeutics.

## 1. Introduction

Ventriculoperitoneal shunts (VP shunts) equipped with a valve to manually switch the shunt function off and a reservoir for the application of intraventricular chemotherapy are an elegant way for intraventricular delivery of therapeutics in patients suffering from a communicating hydrocephalus (hydrocephalus malresorptivus) due to leptomeningeal metastases. We postulate that VP shunts equipped with an on-off valve and a reservoir may be increasingly used in the nearer future in clinical studies exploring local cell-based immunotherapeutics. Therefore, we wanted to determine which shunting devices would be the most appropriate for intraventricular application of therapeutics in patients with leptomeningeal metastases and a communicating hydrocephalus. We decided to screen all adult patients who received a VP shunt with an on-off valve and a reservoir at our institution and performed a retrospective analysis of the clinical course of these patients. We screened the patients– records for reports about problems involving the configuration of the valve settings and during the intraventricular injections and for any shunt-related complications. Furthermore, we investigated the number of intrathecal chemotherapies that were applied using these shunting devices and their clinical outcomes. Intrathecal chemotherapy applied through a reservoir (Rickham or Ommaya reservoir) is usually indicated in patients with leptomeningeal metastases of the diffuse subtype [1]. Alternatively, the use of DepoCyt^®^ obviates the need for intraventricular chemotherapy, as the distribution achieved in the cerebrospinal fluid (CSF) after intralumbar application has been shown to be sufficient as well [2,3]. However, there is no data available on the intrathecal pharmacokinetics of DepoCyt^®^ in patients equipped with a VP shunt [2,4,5]. Rapid drainage through the VP shunt may lead to a loss of efficacy of DepoCyt^®^. In patients suffering from a hydrocephalus due to diffuse leptomeningeal metastases, a connection of the reservoir to a VP shunt is indispensable to drain the CSF and so relieve intracranial hypertension. To ensure a sufficient intrathecal exposure time of the chemotherapeutics, these VP shunts should be furthermore equipped with a valve so that the shunt function can be temporarily switched off. With intraventricular injection of chemotherapeutics, sufficient and uniform concentrations within the subarachnoid space can be achieved [6,7]. There are different types of valves available for this purpose. In type (I), some valves can be switched on and off by pressing a button, while in type (II), the opening pressure can be changed with a specialized device. In the latter type, the opening pressure can be temporarily raised so high that the shunt should not drain cerebrospinal fluid, and therapeutics can be injected fairly reliably through the reservoir into the ventricles [8,9,10]. For the application of intrathecal therapies, those valves that can be switched on and off—type (I)—may be more useful. Shunts equipped with an on-off valve have been shown to allow for effective intrathecal administration of chemotherapeutics in patients suffering from communicating hydrocephalus due to leptomeningeal metastases [11]. Trained practitioners can easily switch the shunt function on and off (“on-off shunts”) without the need for any device-specific equipment. Another advantage is that the configuration of the valve can be checked manually (i.e., after magnetic resonance imaging) on the basis of the position of the button without the need to perform a radiography. Most importantly, when the valve is switched off, the practitioner can be definitively sure that the therapeutics applied do not flow directly through the distal leg of the shunt system into the intraperitoneal space. In this case, it would not be possible to achieve an intrathecal concentration sufficiently high to reach therapeutic efficacy.

Intrathecal chemotherapy is not indicated in patients with an obstructive hydrocephalus because intraventricular injection of chemotherapeutics in these patients fails to result in the uniform distribution of the therapeutic in the CSF necessary for therapeutic efficacy [12]. Another problem in patients with an obstructive hydrocephalus is that the intraventricular concentrations achieved may be locally too high and therefore toxic [13]. Intraventricular chemotherapies are also not effective in patients with a predominantly nodular type of leptomeningeal metastases because chemotherapeutics applied intrathecally can penetrate tissue only a few millimeters deep and do not reach sufficient tissue levels in bigger leptomeningeal metastases [14]. In addition, for many malignancies, more and more targeted therapies are becoming available, which are applied systemically and which are also used in patients with leptomeningeal metastases [15,16]. This has led to a diminished use of intrathecal chemotherapy in general—and VP shunts with on-off valves and a reservoir function in particular—over the course of recent years, at least in our institution.

However, the recent investigation of cellular immunotherapeutic approaches has emphasized new requirements for local treatment modalities in the brain [17,18]. Systemic application of cellular therapeutics may not be an ideal approach for primary brain tumors, brain metastases, and leptomeningeal metastases. Molecular targeted cellular therapeutics may experience difficulties crossing the blood–brain barrier (BBB), adhere in pulmonary capillaries, home to other organs, or trigger systemic toxicity [19]. Similarly, the local treatment of brain tumors poses specific challenges that may be equally difficult to overcome. Primary brain tumors and brain metastases are often located deep within the brain surrounded by functional brain tissue. In particular, glioblastomas are highly infiltrative tumors with tumor cells widely spread in the brain tissue far off from the main tumor masses [20]. Even though tumor cells in leptomeningeal metastases are often distributed over a large area as well, they may be comparatively easy to reach for cellular immunotherapeutics. A recent publication has shown an impressive response of an intrathecal CAR-T cell therapy in a patient suffering from leptomeningeal metastases of a glioblastoma (GB) [21]. Similar approaches probably will be tested in other entities of leptomeningeal metastases from systemic malignancies and primary brain tumors. This is highly relevant as patients who suffer from leptomeningeal metastases often have a dismal prognosis [22,23,24]. A VP shunt with an on-off valve and an injection reservoir may be an ideal device for repeated intrathecal application of cellular immunotherapeutics in patients suffering from a communicating hydrocephalus.

## 2. Results

**Figure 1:**
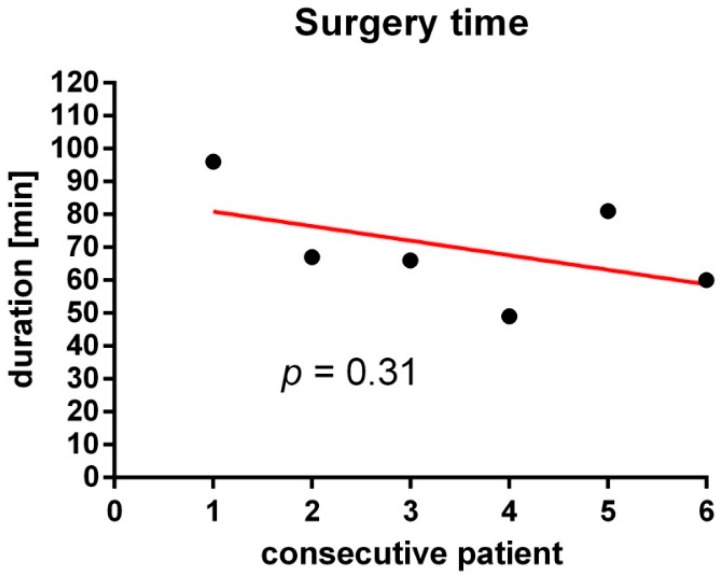
Duration of the shunt implantation procedure of consecutive patients as measured by the time interval from skin incision until skin suture.

No specific difficulties occurred in the implantation of the shunt systems, and there was no perioperative mortality or morbidity. We recorded the duration of the implantation procedure (time interval between the skin incision and the skin suture) to see if there was a learning effect in the neurosurgeons, possibly leading to shorter surgery durations in consecutive patients. The median duration of implantation procedure (time interval between skin incision until skin suture) was 66.5 min (49–96 min). There was a trend for shorter surgery durations; however, it was not significant (*p* = 0.31; Figure 1).

Out of the six patients who received a VP shunt with an on-off valve, intraventricular chemotherapies were subsequently applied in two patients (Table 1). No problems occurred in the configuration of the on-off valve. This can be done easily by oncologist practitioners after a short instruction. In all patients, temporary closure of the shunt system was tolerated, which was tested by switching the shunt function off overnight while the patients were in our in-patient treatment. None of our six patients suffered from symptoms caused by pressure fluctuations in the standing position. This may be probably due to the equipment of our shunt systems with a gravitational unit. In the two patients where an intraventricular chemotherapy was applied, the shunt system was switched off for 24 h after intraventricular application of the chemotherapy. Both patients tolerated this temporary closure of the shunt system without showing clinical signs of a raised intracranial pressure. In four patients, the intrathecal chemotherapy was omitted due to the development of a nodular type of leptomeningeal metastases or rapid clinical deterioration.

Patient 4 received a total of nine intraventricular injections of methotrexate (MTX). Due to a lack of response, the intrathecal chemotherapy was switched to thiotepa. After nine weekly intraventricular injections, a durable remission of CSF parameters was reached. In follow-up CSF examinations, CSF cytologies repeatedly did not evidence malignant cells, an elevated cell number, or elevated lactate concentrations. Therefore, the intraventricular therapy was stopped. However, 34 weeks after shunt implantation, the leptomeningeal metastases relapsed. Intraventricular thiothepa injections were resumed, but by then they were no longer effective. After a total of eight thiothepa injections, the therapy was switched to a systemic therapy with Gefitinib. As the patient did not respond to Gefitinib either, the therapy was changed after eight weeks to a pulsatile scheme with Erlotinib, which had a good effect. After 68 weeks under therapy with Erlotinib, the patient progressed and succumbed to her disease 116 weeks after shunt implantation (Table 2).

**Figure 2:**
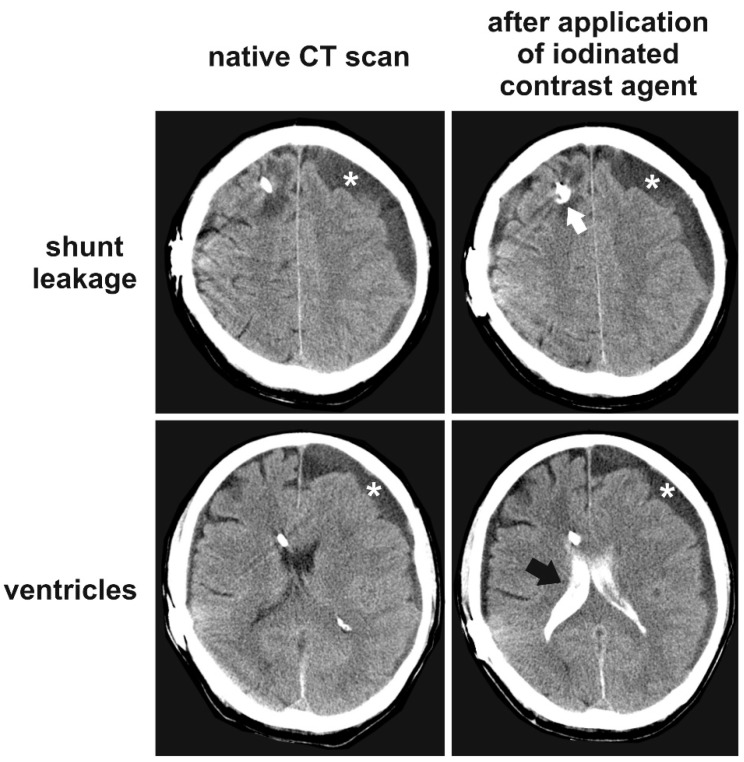
Shunt leakage in patient 6. This patient developed severe headaches immediately after the first intraventricular injection of thiotepa. The ventricles were promptly filled with the iodinated contrast enhancer after injection through the shunt catheter (black arrow). However, we detected a shunt leakage shortly after the entry point of the shunt catheter in the brain (white arrow). The hypodense area over the left hemisphere (white asterisks) is a pre-existing chronic subdural hematoma.

One patient experienced complications involving the VP shunts. Patient 6 developed strong headaches immediately after the first intaventricular injection of thiotepa through the shunt system. The total volume injected was 11 mL (5 mL thiotepa and then 6 mL of 0.9% sodium chloride solution to flush the proximal shunt system). During the injection procedure, we did not observe an elevated resistance of the shunt system. We suspected a shunt dysfunction and performed computertomography (CT) imaging of the brain before and after injecting iodinated contrast enhancer through the shunt system. The volume injected for the radiography was similar to the volume injected for the application of the chemotherapy. We injected a volume of 9 mL (3 mL contrast enhancer and then 6 mL of 0.9% sodium chloride solution). The shunt was again easily passable without posing resistance to the injection, and the ventricles were promptly filled by the contrast enhancer. Notably, the headaches did not deteriorate during the injection of the contrast enhancer. Nonetheless, a shunt leakage near the insertion point of the shunt catheter in the brain was detected (Figure 2). The patient described the headaches as bilateral equally pressing in the forehead and lasting for approximately 30 h. The headaches were responsive to both ibuprofen and piritramide. Due to the long duration of the headaches, the relatively low volume of the leakage, the patency of the shunt system, and the missing deterioration during the injection of the contrast enhancer, we suspect that the headaches were triggered by a toxic irritation of the meninges rather than compression of adjacent brain tissue. As the patient did not present with the complete triad of meningism (only headaches; no nuchal rigidity and no photophobia) this meningeal irritation seemed to be local. We estimate the possibility of the shunt catheter leakage being caused by the injection of the chemotherapy itself as rather unlikely because we injected the intrathecal chemotherapy very slowly and without inducing much pressure in the shunt system. We suspect that the leakage may have occurred unnoticed during the implantation procedure. In addition, we cannot rule out a preexisting hidden material defect in the shunt catheter.

The intrathecal chemotherapy was therefore omitted and a whole brain radiotherapy was instead applied. The shunt system was not replaced because the drainage function of the shunt system was not impaired.

Four out of the six patients eventually did not receive any intrathecal chemotherapy. Patients 1, 2, and 5 showed an increasingly nodular type of leptomeningeal metastasis and therefore the initially planned intrathecal therapy was skipped. Patient 1 received a systemic therapy with Afatinib. Patients 2 and 5 received a whole brain radiation therapy. In patient 3, the intrathecal chemotherapy was omitted due to a rapid clinical decline and the patient therefore received best supportive care (BSC). 

**Figure 3:**
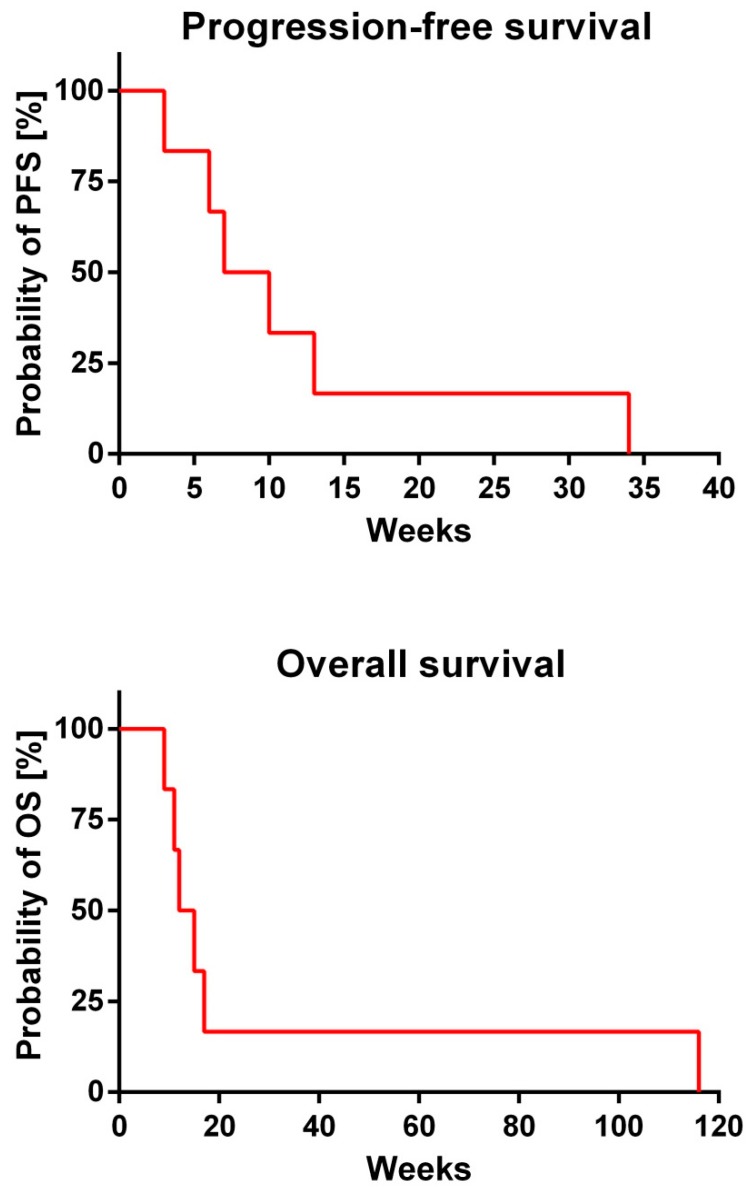
Progression-free survival (PFS) and overall survival (OS) after shunt implantation.

The median progression-free survival (PFS) of the patients reported in this series was 8.5 weeks and the median overall survival (OS) was 13.5 weeks (Figure 3).

## 3. Discussion

Similarly to the results reported by Lin et al. [11], we have shown that shunts equipped with an on-off valve and a reservoir function allow for uncomplicated and effective intraventricular administration of chemotherapies in patients with leptomeningeal metastases. We used shunt systems that differed from the ones used by Lin et al. [11] as they were additionally equipped with gravitational units. These shunts are reliable tools for injecting therapeutics intraventricular without running the risk of injecting intraperitoneally. Avoiding unintentional intraperitoneal injections and ensuring intraventricular injections is of high significance when applying intraventricular chemotherapies. For effective intrathecal treatment of leptomeningeal metastases, achieving sufficient concentrations over prolonged time intervals is paramount. Intrathecal application of chemotherapeutics through repeated lumbar punctures is only effective if prolonged-release formulations are used because only then can sufficient intraventricular concentrations be achieved [4,12]. Similarly, an effect of intraventricular therapy should only be expected if the chemotherapeutics applied are not drained immediately through a ventriculoperitoneal shunt [25]. Most importantly, VP shunts equipped with an on-off valve and a reservoir serve two different requirements in patients with communicating hydrocephalus due to leptomeningeal metastases—(I) to enable the drainage of CSF and (II) to establish a reliable route of intraventricular access for chemotherapeutics.

In the setting of cellular immunotherapies intended for intrathecal application, this may be of an even higher significance as systemic toxicities may be even more of a concern. Shunts with on-off valves are very easy to handle, including in the setting of a neuro-oncology outpatient-department. There is no need to provide specialized equipment to change the opening pressure of shunt valves. Practitioners who are not familiar with this specialized equipment are nevertheless easily able to switch the shunt function on and off after a short one-time instruction without major problems.

The complication of shunt leakage that occurred in one patient of this series is a typical and common shunt complication [26,27]. The causation of the shunt leakage in patient 6 is not entirely clear. It may have occurred unnoticed during the implantation procedure or may be the result of a hidden material defect. We did not experience problems with the function of the programmable differential pressure units or the gravitational units. We purposely positioned the on-off valve between the Rickham reservoir and the programmable differential pressure units (and not distal to the pressure units or the gravitational units). We consider this sequence as highly important to protect these very vulnerable components from pressure fluctuations during the injection procedures. However, the number of patients of our series is too low to draw any conclusion about the frequency of complications, which is a main limitation of this study. It is therefore also not possible to determine if the rate of complications is different compared to patients who received shunts not equipped with on-off valves. However, this patient series still demonstrates the usefulness, in principle, of VP-shunts equipped with on-off valves for the intrathecal administration of chemotherapies in patients with a communicating hydrocephalus due to leptomeningeal metastases.

Four out of six patients in our cohort finally did not receive an intraventricular chemotherapy because they developed a nodular distribution pattern of leptomeningeal metastases or declined rapidly. This considerable high proportion was to be expected due to the dismal prognosis of leptomeningeal metastases in most malignancies, with a median survival of only several weeks. The survival times recorded here (median PFS of 8.5 weeks and median OS of 13.5 weeks) are in accordance with the data documented in the literature [28,29,30,31,32].

We are currently performing a phase I clinical trial testing the safety and tolerability of NK-92 cells ectopically expressing a chimeric antigen receptor (CAR) targeting HER2 in patients with recurring HER2 positive GB (CAR2BRAIN study) [33,34]. These CAR-NK cells are injected into the resection wall during relapse surgery. In an escalation cohort, these cells will be subsequently repeatedly injected through a conventional Rickham reservoir into the resection cavity. An intraventricular injection of the CAR-NK cells is not planned as part of the study. However, an intraventricular injection of CAR-NK cells may be an option for patients with leptomeningeal metastases and communicating hydrocephalus. Brown et al. have shown an impressive effect of intraventricular application of CAR-T cells targeted against IL13Rα2 in a patient suffering from a GB and leptomeningeal metastases. Several leptomeningeal metastases of the cerebrum and the spine regressed [21]. To avoid systemic toxicity as far as possible, an intraperitoneal injection of CAR-NK cells should be ruled out. A VP shunt equipped with a reservoir function and an on-off valve should therefore be an appropriate solution for the intraventricular application of cellular immunotherapeutics in patients with a communicating hydrocephalus due to leptomeningeal metastases.

## 4. Patients and Methods

**Figure 4:**
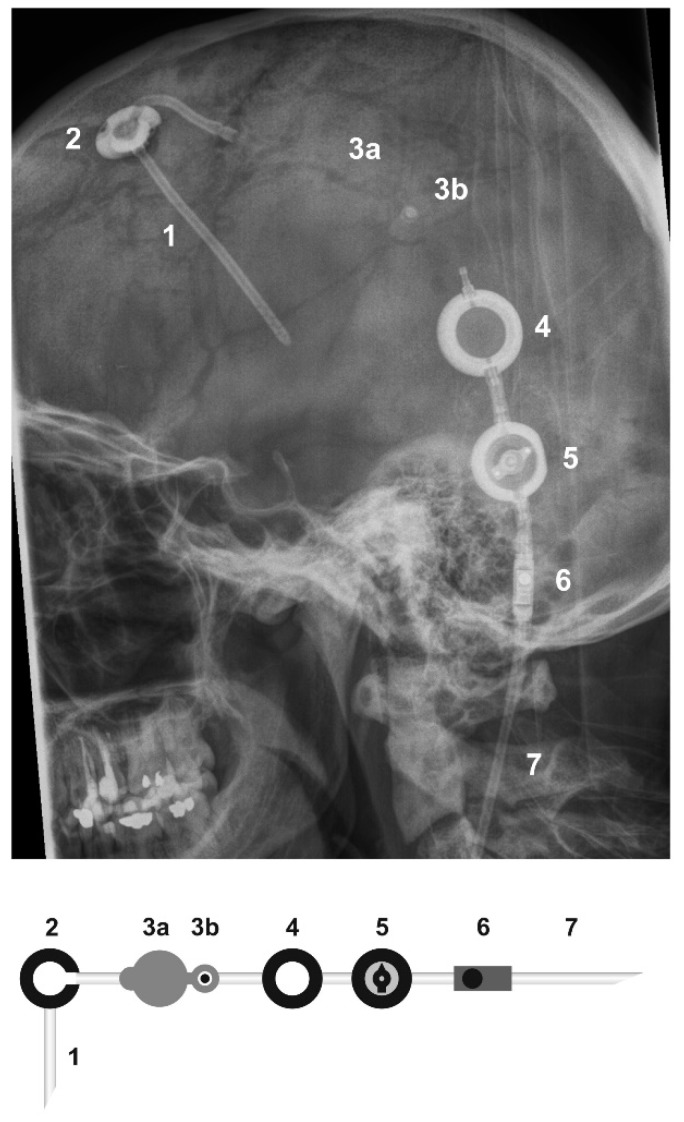
Radiography immediately after shunt implantation in patient 1 and scheme depicting the arrangement of the shunt components. 1 = proximal ventricular catheter; 2 = Rickham reservoir; 3a = on-off valve; 3b = button of the on-off valve; 4 = control reservoir; 5 = programmable differential pressure unit; 6 = gravitational unit; 7 = distal peritoneal catheter.

We report on six consecutive adult patients who had a VP shunt with an on-off valve implanted at the Department of Neurosurgery at the Goethe University Hospital between April 2008 and July 2017. All patients received an Aeskulap-Miethke proGAV^®^ shunt (FV435T) with an additional Integra^®^ on-off flushing reservoir (NL8500150) positioned in the proximal leg of the shunt system (Figure 4). Similarly to the on-off shunts reported by Lin et al. [11], these shunts were composed of a reservoir connected in series with an on-off valve, a programmable differential pressure unit, and a distal peritoneal shunt. However, our shunt systems were additionally equipped with a control reservoir to allow for manually checking if the shunt system is passable as well as a gravitational unit.

First, we screened our database for all adult patients (at least 18 years old) with brain metastases, leptomeningeal metastases, or a primary brain tumor who had a VP shunt implanted. We excluded pediatric patients from our analysis because they had received a different shunt system. Furthermore, pediatric patients typically received their VP shunt in a different therapeutic situation shortly after initial diagnosis of pediatric brain tumors to allow for the intrathecal application of intrathecal chemotherapies as part of combination chemotherapy schemes.

Then, we singled out six patients—from the 126 patients who fulfilled these criteria—whose shunt was equipped with an on-off valve. We analyzed the patient records for complications and the amount of intrathecal chemotherapies applied. All six patients suffered from a communicating hydrocephalus caused by leptomeningeal metastases of a systemic malignancy (Table 3). All patients had a diffuse subtype of leptomeningeal metastases without nodular leptomeningeal contrast enhancement at the time of shunt implantation. In all patients, the diagnosis of leptomeningeal metastases was established by CSF cytology. The duration of the implantation procedure was extracted from the surgical record. The possible significance in reduction of the duration of the implantation procedure in consecutive patients was calculated with the simple linear regression model.

Our institutional review board approved this retrospective study, and all patients or their legal guardian gave their written consent for scientific work with clinical data, including radiological imaging (ethics committee at the University Hospital; reference number 04/09-SNO-03-2018).

## 5. Conclusions

Ventriculoperitoneal shunts equipped with a valve to switch the shunt function on and off are a useful and reliable tool for the intraventricular administration of therapeutics in patients suffering from a hydrocephalus due to diffuse leptomeningeal metastases. These shunts may also be used for the intraventricular application of cellular immunotherapeutics.

## Figures and Tables

**Table 1:** Intrathecal chemotherapy.

Pat. No.	Intrathecal Chemotherapy Applied	Reasons for Omitting Intrathecal Chemotherapy	Complications	Number of Intraventricular Injections
1	None	Nodular type of meningeal metastases	None	0
2	None	Nodular type of meningeal metastases	None	0
3	None	clinical deterioration	None	0
4	MTX, thiotepa	-	None	9 MTX 17 thiotepa
5	None	nodular type of meningeal metastases	None	0
6	Thiotepa	-	Shunt leakage	1

MTX = methotrexate; Pat. No. = patient number.

**Table 2:** Progression-free and overall survival.

Pat. No.	PFS (Weeks)	OS (Weeks)
1	7	12
2	10	11
3	3	9
4	34	116
5	6	15
6	13	17

OS = overall survival after shunt implantation; PFS = progression-free survival after shunt implantation.

**Table 3:** Patient characteristics.

Pat. No.	Age at Shunt Implantation	Sex	Underlying Disease
1	33 years	F	NSCLC
2	50 years	F	breast cancer
3	57 years	M	transitional cell carcinoma
4	60 years	F	NSCLC
5	63 years	F	breast cancer
6	70 years	M	prostate cancer

F = female; M = male; NSCLC = non-small-cell lung cancer; Pat. No. = patient number.
